# Metabolic Inflammation-Differential Modulation by Dietary Constituents

**DOI:** 10.3390/nu8050247

**Published:** 2016-04-27

**Authors:** Claire L. Lyons, Elaine B. Kennedy, Helen M. Roche

**Affiliations:** Nutrigenomics Research Group, UCD Conway Institute of Biomolecular and Biomedical Research and UCD Institute of Food and Health, University College Dublin, Belfield, Dublin 4, Ireland; claire.lyons.2@ucdconnect.ie (C.L.L.); elaine.kennedy.1@ucdconnect.ie (E.B.K.)

**Keywords:** nutrition, metabolic-inflammation, diet, insulin resistance, fatty acids, adipose tissue, liver, muscle, pancreas

## Abstract

Obesity arises from a sustained positive energy balance which triggers a pro-inflammatory response, a key contributor to metabolic diseases such as T2D. Recent studies, focused on the emerging area of metabolic-inflammation, highlight that specific metabolites can modulate the functional nature and inflammatory phenotype of immune cells. In obesity, expanding adipose tissue attracts immune cells, creating an inflammatory environment within this fatty acid storage organ. Resident immune cells undergo both a pro-inflammatory and metabolic switch in their function. Inflammatory mediators, such as TNF-α and IL-1β, are induced by saturated fatty acids and disrupt insulin signaling. Conversely, monounsaturated and polyunsaturated fatty acids do not interrupt metabolism and inflammation to the same extent. AMPK links inflammation, metabolism and T2D, with roles to play in all and is influenced negatively by obesity. Lipid spillover results in hepatic lipotoxicity and steatosis. Also in skeletal muscle, excessive FFA can impede insulin’s action and promote inflammation. Ectopic fat can also affect pancreatic β-cell function, thereby contributing to insulin resistance. Therapeutics, lifestyle changes, supplements and dietary manipulation are all possible avenues to combat metabolic inflammation and the subsequent insulin resistant state which will be explored in the current review.

## 1. Introduction

**Figure 1:**
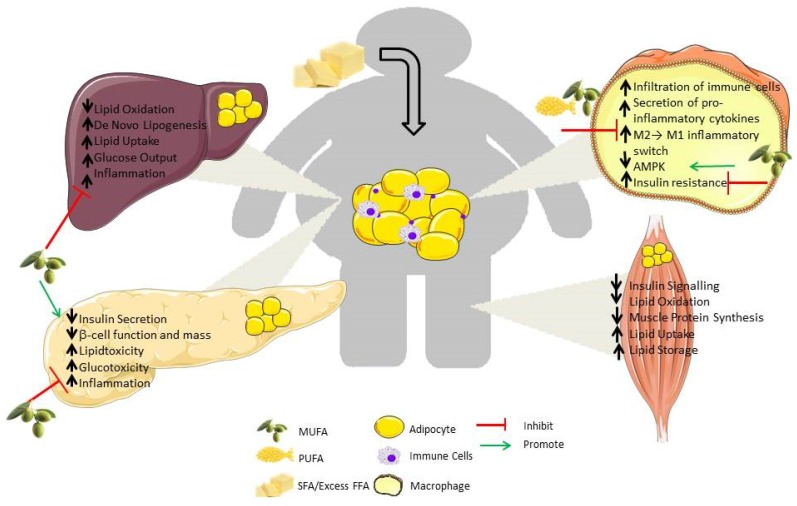
Metabolic-inflammation: Implication of free fatty acid (FFA) driven insulin resistance on the major metabolic organs. As adipose tissue expands due to excess nutrients, immune cells infiltrate causing chronic low-grade inflammation and metabolic changes. Ectopic lipid spill-over from the adipose to the liver, muscle and pancreas results in glucotoxicity and lipotoxicity. All of these disruptions culminate in impaired insulin signaling, dysregulated glucose homeostasis and development of insulin resistance and type 2 diabetes (T2D). Differential modulation by fatty acids occurs, whereby saturated fatty acids (SFA) exacerbate the situation, while monounsaturated fatty acids (MUFA) and polyunsaturated fatty acids (PUFA) reduce this metabolic inflammatory state. (This figure was prepared using the Servier medical art website [4].

Obesity is caused by chronic energy imbalance, wherein calorie intake exceeds calorie expenditure, leading to weight gain over time. Overweight is defined as a body mass index (BMI) greater than 25 kg/m^2^, with a BMI greater than 30 kg/m^2^ being defined as obese. The obese state is associated with chronic low-grade inflammation within the metabolic tissues, often referred to as metabolic-inflammation, or “meta-inflammation” [1]. Several events trigger and propagate this sub-acute, chronic inflammatory state which is characteristic of obesity. Exposure to free fatty acids (FFA) which initiate inflammatory signaling, immune cell infiltration and a shift in inflammatory cell populations contribute to inflammation in metabolic tissues. Under normal weight conditions, adipose tissue has the capacity to store FFA effectively. However, in the obese state, the storage capacity of adipose tissue is exceeded. In this case FFA “spill over” and accumulate in metabolic tissues such as skeletal muscle, liver and pancreas causing lipotoxicity [2]. Excess FFA in turn can activate inflammatory pathways and impair normal cell signaling within immune cells, adipose tissue, liver and muscle, causing cellular dysfunction [3]. Consequently, metabolic disorders such as insulin resistance and type 2 diabetes (T2D) can develop, as illustrated in Figure 1.

Adipose tissue is the main storage organ involved in energy homeostasis and it also functions as an endocrine organ. The adipose tissue is composed of adipocytes and the smaller, albeit equally important stromal vascular fraction (SVF), which contains a range of immune cells, fibroblasts and pre-adipocytes. Other organs that play a vital role in metabolism are liver, muscle and the pancreas. The liver is the main site of glucose production and the muscle the main site of glucose disposal. Critical to the regulation of glucose uptake is the hormone insulin, produced and secreted by the pancreas. Obesity acts as a nutritional stressor not only modifying adipose metabolism, but also by substantial lipid spill over into the other metabolic organs, the culmination of which leads to insulin resistance and T2D. In obesity, the immune cell compartment in adipose plays a critical role in the development of metabolic disease. The immune system is pivotal to this whole process and the link between nutrition (with a specific focus of fatty acid composition), inflammation and metabolism will be the focus of this review.

## 2. Mechanisms of Metabolic-Inflammation in Adipose Tissue

### 2.1. Immune Cell Infiltration

Immune cells are paramount to the initiation and propagation of metabolic-inflammation, with the adipose tissue acting as the initial site of obesity-induced inflammation. With increasing weight, the adipose tissue expands to deal with the need to store excess nutrients. Adipose tissue expansion can occur in two ways, with hyperplasia or hypertrophy, an increase in adipocyte number or size, respectively. Hypertrophic obesity is associated with expansion of existing adipocyte size, wherein the morphology shows greater adipocyte volume. Hypertrophic obesity is usually associated with insulin resistance. Hyperplasic adipose is associated with insulin sensitivity. The adipocytes increase in number, and are therefore better equipped to deal with the demand for excess energy/lipid storage. Enhanced adipogenesis, the process wherein adipocytes are formed, is associated with hyperplasic adipose. Adipogenesis is impeded by inflammatory mediators, such as caspase-1 and interleukin-1 beta (IL-1β) [5]. Casp1−/− animals fed a high-fat diet (HFD) display smaller adipocytes, with reduced adipose mass. In addition, the composition of the HFD may affect adipose morphology. We showed that a saturated fatty acid (SFA) enriched HFD, derived from palm oil, induced hypertrophic adipose. In contrast, a monounsaturated fatty acid (MUFA) enriched HFD derived from oleate was associated with a hyperplasic adipocytes and improved insulin sensitivity [6]. This highlights the concept that adipose morphology may confer differential functionality.

Resident mast immune cells help facilitate growth through extracellular remodeling [7]. Along with the help of adipose tissue macrophages (ATM) and endothelial cells [8], mast cells provide increased angiogenesis to ensure the tissue has adequate vasculature and blood supply to complete the expansion process [9]. Adipocytes can become stressed with the increased workload, leading to obesity-induced adipocyte cell death, a process correlated to increased adipocyte size [10]. Within this context, the initial infiltration of immune cells from the periphery into the adipose tissue [11] is thought to be a protective mechanism. Their role is to clear the necrotic adipocytes, as indicated by their localization in crown-like structures (CLS), surrounding these cells in a syncytia containing the scavenged free lipid of the adipocyte [8,12].

Adipose tissue secretes a range of chemokines, which supports its role as an endocrine organ. This “immuno-phenotype” reflects the infiltration and proliferation of several immune cells. Monocyte chemoattractant protein 1 (MCP-1) recruits immune cells and acts as a beacon leading their way to sites of infection and inflammation [13]. Immune cell number is positively correlated with increasing adiposity [11]. Increasing macrophage and T cell infiltration is also observed in the skeletal muscle [14] of obese humans and in the liver [15] of mice with diet-induced obesity (DIO). Immune cell infiltration is one of the earliest events observed in the obese setting, with pro-inflammatory gene expression preceding hyperinsulinemia [12]. Nishimura and colleagues demonstrated that pro-inflammatory CD8+ T cells, followed by macrophages, entered the adipose tissue as early as four weeks on a HFD. Adipose tissue itself can activate T cells [8], which are necessary for the migration, differentiation and activation of macrophages. Upon HFD feeding, the antigen presenting dendritic cells (DC) infiltrate the adipose tissue and display an increased activation state [16]. There is also a subsequent decrease in the numbers of anti-inflammatory cells, such as T regulatory (Treg) and anti-inflammatory macrophages [8,17]. As the immune cells do not have a real target to overcome, they remain in the adipose tissue long after they are needed, contributing to the chronic low-grade inflammation, one of the characteristic hallmarks of metabolic dysregulation. Weight loss has been shown to reduce immune cell number within this depot [18], indicating that obesity itself is one of the main drivers of immune cell infiltration.

### 2.2. Adipose Tissue Macrophages (ATM)

ATMs are vital components of metabolic-inflammation. The ATM population increases from 10%–15% to 45%–60% with the progression of obesity [11]. Macrophages were once thought to have either an M1 pro-inflammatory or M2 anti-inflammatory phenotype [19], but are now believed to exist across a spectrum. With obesity existing, resident immune cells in adipose tissue undergo a phenotypic switch from M2 to M1, resulting in a pro-inflammatory immuno-phenotype. The nature of macrophage polarization can be affected by fatty acid composition. For example, saturated fatty acids (e.g., palmitic acid (PA)) activate pro-inflammatory M1 genes (tumor necrosis factor alpha (Tnfα), interleukin-6 (Il6)). In contrast, MUFA (e.g., palmitoleate (PO)), activates and promotes the pro-resolving M2 (arginiase-1 (Arg1), interleukin-10 (Il10)) phenotype [20]. Interestingly, Kratz *et al*. identified a distinct population of metabolically activated macrophages (MMe), following palmitate, glucose and insulin challenge [21]. Mme macrophages displayed M2 markers of lipid metabolism, adenosine triphosphate (ATP)-binding cassette transporter (ABCA1), cluster of differentiation 36 (CD36) and perilipn 2 (PLIN2), They also secreted similar levels of pro-inflammatory cytokines to that of M1 classical macrophages. However, typical M2 markers, mannose receptor (CD206), and M1 cell surface markers, cluster of differentiation (CD38), were absent in the MMe. ATM from obese human subjects and mice had a similar profile to that of MMe demonstrating their existence *in vivo* and a positive correlation with increased adiposity. Peroxisome proliferator-activated receptor gamma (PPARγ) and sequestome-1 (p62) were responsible for promotion of the cell surface receptors of MMe and restrict the secretion of pro-inflammatory cytokines, such as IL-1β. M2 macrophages are responsible for maintaining the adipose tissue in an insulin sensitive state, through the anti-inflammatory action of IL-10 and signal transducer and activator of transcription 3 (STAT3) pathways [19], whereas M1 secrete pro-inflammatory cytokines contributing to insulin resistance. Also, our work demonstrated that the immuno-phenotype of ATM can differ in response to HFDs, despite equal ATM numbers. Adipose cytokine secretion was markedly attenuated despite a HFD in IL-1RI−/− mice with equivalent ATM number, compared to wild-type (WT) [22]. Hence, both the ATM numbers and the nature of the metabolic agonist can define the nature and functionality of ATM in obesity.

### 2.3. Differential Modulation of Inflammatory Mediators in Obesity

Immune cell infiltration generates inflammatory signals within the metabolic tissues, which disrupt insulin signaling. Hotamisligil and colleagues first demonstrated that within obesity, TNF-α was a key player in insulin resistance [23]. Nutrient and pathogen sensing pathways share common signaling mechanisms within the cell. Toll-like receptors 2 and 4 (TLR2/4) are cell surface pathogen recognition receptors (PPR) through which SFA and lipopolysaccharide (LPS) activate nuclear factor kappa B (NF-κB) transcription, to elicit pro-inflammatory cytokine secretion [24,25]. LPS- and PA-induced cytokine secretion is not observed in TLR4−/− mice. TNF-α reduces glucose transporter 4 (GLUT4) translocation [23] reducing glucose uptake and affecting insulin signaling by inhibiting the tyrosine phosphorylation (pTyr) of the insulin receptor [26], necessary for its action. FFA-activation of TLR4 reduces both glucose homeostasis and insulin sensitivity [25].

PA stimulation promotes macrophage I kappa B alpha (IκBα) degradation, janus kinase (JNK) phosphorylation, with TNF-α and IL-6 secretion *in vitro*. Stimulation with both TNF-α and IL-1β elicited a greater induction of NF-κB activity than either cytokine alone. Furthermore, the loss of synergy effect between TNF-α and IL-1β was found in IL-1RI−/− explants, with reduced IL-6 secretion as the readout [22]. WT and Tnfa−/− mice injected with IL-1β had similar IL-1β induction, with a greater induction of TNF-α and subsequent insulin resistance in the WT mice, demonstrating the effect of TNF-α induction by IL-1β [27]. *In vivo*, feeding a HFD also amplifies IL-1β inflammation, with PA specifically shown to activate the nod-like receptor protein (Nlrp3) inflammasome [6,27]. Priming first occurs through TLR4 signaling to produce the immature pro-IL-1β form. Further processing, through the formation of the Nlrp3 inflammasome, produces caspase-1, which cleaves IL-1β into its mature and active form. IL-1 has multiple biological functions including fever, inflammation and innate immune responses, but, in this context, its impact upon insulin resistance and adipose metabolism is noteworthy [22]. Increased expression of Nlrp3 components are correlated with adiposity in DIO, genetic models of obesity (db/db and ob/ob) and obese humans [5,6,28,29]. The SVF is the primary source of IL-1β in adipose tissue [6,28]. Adipose tissue explants from IL-1RI−/− HFD mice release less IL-6, TNF-α and IL-1β compared to their WT obese counterparts [22]. IL-1β can negatively influence insulin signaling and subsequent glucose uptake, thereby demonstrating its role in HFD-induced insulin resistance [16,22,27,29]. Also, DIO Nlrp3−/− mice display increased subcutaneous adipose tissue M2 transcripts (Il-10, Arg1) with increased visceral adipose tissue M1 transcripts (Tnfa) [28]. The composition of fat affects Nlrp3 and IL-1β activation, indicating an interplay between Nlrp3 and IL-1βactivation in response to dietary fat. Mice fed a MUFA-HFD had reduced pro-IL-1β, with lower IL-1β secretion compared to SFA-HFD [6]. This Nlrp3 and IL-1β effect of SFA-HFD was significantly reduced when the Nlrp3 inflammasome was inhibited, indicating a specific role. Caloric restriction was capable of reducing the expression of inflammasome components indicating that obesity is critical to their induction [28].

PA can increase reactive oxygen species (ROS) production and IL-1β, which were no longer produced when either ROS or nicotinamide adenine dinucleotide phosphate (NADPH) oxidase were inhibited. Furthermore, activation of AMPK with 5′-aminoimidazole-4-carboxamide ribonucleotide (AICAR) attenuated the LPS- and PA-induction of ROS, pro-IL-1β and IL-1β [27]. In addition, modulation of Nlrp3 and IL-1 by different HFD was also associated with reciprocal regulation of insulin sensitivity and adipogenesis, probably mediated by AMPK [6]. Ceramide stimulation of bone marrow derived macrophages (BMDM) with LPS induced IL-1β and caspase-1 but was ablated in Nlrp3−/− cells [28]. In mice with deletions in Nlrp3−/−, Pycard−/− (apoptotic speck protein containing a caspase recruitment domain, known as ASC, encoded by the PYCARD gene) and Casp1−/−, PA fails to induce both pro-IL-1β and IL-1β, with no effect on TNF-α, in LPS primed BMDM, DC and peritoneal macrophages. In contrast, oleic acid (OA) showed no enhancement of IL-1β, IL-6 and TNF-α secretion in conjunction with LPS stimulation [27].

**Figure 2:**
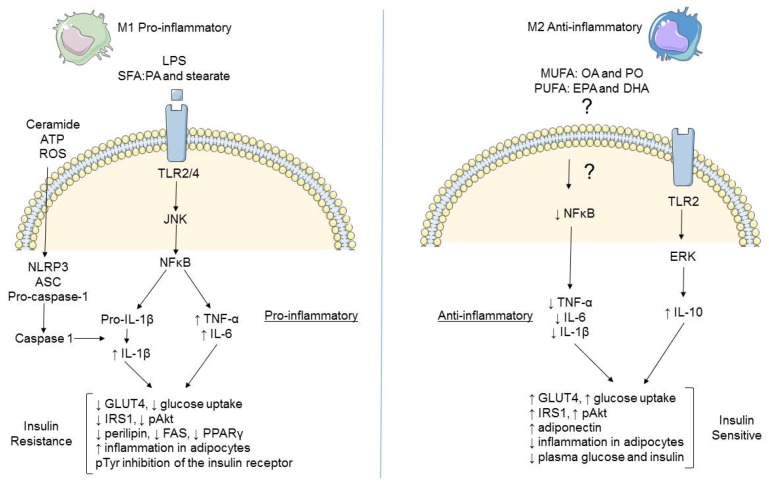
Inflammatory pathways in M1 and M2 macrophages. M1 pro-inflammatory macrophages are induced by saturated fatty acids (SFA) and lipopolysaccharide (LPS) to generate pro-inflammatory signaling through nuclear factor kappa B (NFkB) to produce tumour necrosis factor alpha (TNF-α) and interleukin-6 (IL-6). Subsequent stimulation by ceramides, adenosine triphosphate (ATP) or reactive oxygen species (ROS) leads to assembly of the nod-like receptor (Nlrp3) inflammasome and processing of pro-interleukin-1 beta (IL1β) to active IL-1β through cleavage by caspase-1. Pro-inflammatory cytokines negatively impact glucose homeostasis and insulin signaling, resulting in insulin resistance in neighbouring cells. M2 anti-inflammatory macrophages are induced by monounsaturated fatty acids (MUFA) and polyunsaturated fatty acids (PUFA) acting via receptors, which are currently unidentified, with increased interleukin-10 (IL-10) secretion along with a reduction in pro-inflammatory markers. This results in improved insulin sensitivity and a less inflammatory environment. TLR = toll-like receptor, ASC = apoptosis like speck protein, GLUT4 = glucose transporter type 4, IRS = insulin receptor substrate, FAS = fatty acid synthase. PPARγ = peroxisome proliferator activated receptor gamma, OA = oleic acid, PO = palmitoleate, EPA = eicosapentaenoic acid, DHA = docosahexaenoic, ERK = extracellular regulated kinase. (This figure was prepared using the Servier medical art website [4]).

The FFA stearate also phosphorylates mitogen activated protein kinase (MAPK), with extracellular signal-regulated kinase (ERK) and JNK activation to elicit macrophage activation [30]. Conversely, the long chain *n*-3 polyunsaturated fatty acids (LC *n*-3 PUFA), eicosapentaenoic acid (EPA) and docosahexaenoic acid (DHA) do not promote inflammation, characterized by TNF-α, IL-1β and IL-6 responses in LPS-stimulated macrophages [31]. Pre-treatment with DHA prevented PA-induced inflammation [27]. There is some evidence to suggest that DHA may have more potent anti-inflammatory potential, compared to EPA [31]. Also, this anti-inflammatory effect may impact upon insulin signaling. Co-culture of DHA- and LPS-treated macrophages with 3T3-L1 adipocytes resulted in improved insulin sensitivity with increased GLUT4 expression and increased glucose uptake, thereby illustrating the differential modulation of inflammation and insulin sensitivity by various fatty acids [32], as described in Figure 2.

### 2.4. Integration of Metabolism and Immune Responses

**Figure 3:**
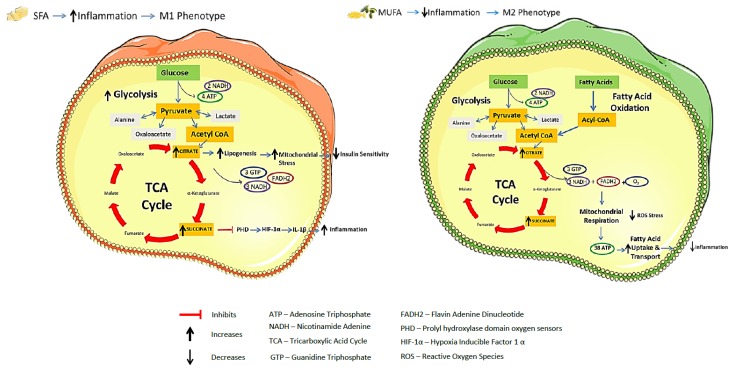
Integration of metabolism and immune responses. Obesity and SFA drive an M1 pro-inflammatory phenotype in macrophages which favours glycolysis for ATP generation and leads to TCA cycle fragmentation with a break at the succinate dehydrogenase step. This break in the TCA cycle results in increased succinate and citrate accumulation. Citrate accumulation impedes insulin sensitivity by increasing lipogenesis and mitochondrial stress. Succinate inhibits PHD, stabilizing HIF-1α leading to activation of the pro-inflammatory cytokine IL-1β. While MUFA and PUFA drive an anti-inflammatory M2 phenotype which favours the more energy efficient process of oxidative phosphorylation, with increased fatty acid oxidation and glutamine metabolism. (This figure was prepared using the Servier medical art website [4]).

Both the immune and metabolic systems share similarities in terms of maintaining the homeostatic balance during times of health, and adapting during times of stress (e.g., inflammation or obesity). Increased metabolism is required to provide sufficient energy to mount a successful immune response [33]. Metabolism requires the use of ATP as the body’s fuel derived from nutrients, as illustrated in Figure 3. Glycolysis utilizes glucose as its substrate to generate pyruvate as the final product, with four molecules of ATP and two molecules of nicotinamide adenine (NADH) formed in the process. Pyruvate can then enter the tricarboxylic acid cycle (TCA) resulting in production of three guanidine triphosphate (GTP), flavin adenine dinucleotide (FADH2) and three molecules of NADH. Amino acids can also feed into the TCA cycle as can fatty acids, through the process of β-oxidation. The FADH2 from the TCA cycle combines with NADH and oxygen, to initiate the electron transport chain, within the mitochondria, culminating in the activation of ATP synthase and finally up to 38 molecules of ATP, making this process of oxidative phosphorylation a more efficient process than glycolysis. Alternatively, pyruvate can be converted to lactate instead of entering the TCA cycle. Metabolism is influenced by substrate availability and dictated by the diet. In addition to the phenotypic switch that macrophages undergo during inflammation and obesity, a switch in metabolism also occurs in these cells, despite the availability of relevant substrates, a phenomenon known as the Warburg effect [34]. Neutrophils and macrophages rely on aerobic glycolysis for ATP generation, despite the fact that oxidative phosphorylation is more efficient [35]. This results in less glucose being present and the resulting pyruvate being converted to lactate instead of entering the TCA and oxidative phosphorylation pathways [34]. Obesity and SFA in particular, induce the pro-inflammatory M1 phenotype as discussed previously, while MUFA and PUFA induce the anti-inflammatory M2 macrophage. Transcriptional metabolic profiling of macrophage polarization revealed different metabolic pathways in use for M1 and M2 activation, thus as nutrients influence the inflammatory state, this in turn determines the type of metabolism used. M1 pro-inflammatory macrophages adopt this metabolic reprogramming [36], with TCA cycle fragmentation, and a break at the succinate dehydrogenase step, in conjunction with increased glycolysis [37]. Inflammatory stimuli such as LPS increase macrophage glycolysis by activating pyruvate kinase M2 (PKM2), the rate limiting kinase in this pathway [36]. When the Warburg effect is initiated in LPS stimulated BMDM, there is greater utilization of glycolysis, the TCA cycle is effectively broken and the intermediates succinate and citrate accumulate [38]. Succinate acts as a pro-inflammatory metabolic signal, inhibiting prolyl hydroxylase domain oxygen sensors (PHD), thereby, stabilizing hypoxia inducible factor 1-α (HIF-1α) [36,38]. HIF-1α itself leads to activation of the pro-inflammatory cytokine, IL-1β [38], a well-known player in HFD-induced inflammation and insulin resistance [22]. Citrate in turn promotes lipogenesis, which in turn also promotes inflammation. Thus, elevated circulating succinate and citrate levels in metabolic disease reflect integrated dysregulation of metabolism and inflammation [39,40].

In contrast, alternative M2 macrophages continue to use oxidative phosphorylation [41] and are involved in glutamine metabolism [42]. T helper 2 cells (Th2)-derived interleukin-4 (IL-4) induces alternative anti-inflammatory activation of macrophages. The coactivator peroxisome proliferator-activator receptor gamma, coactivator 1-beta (PGC-1β), is responsible for the transcription of genes involved in fatty acid oxidation, fatty acid transport and uptake. All of these are not only increased in the alternative macrophage, but are inversely related to the pro-inflammatory secretome of these macrophages. Conversely, LPS and interferon gamma (IFNγ) treated macrophages display reduced fatty acid oxidation. Inhibitors of fatty acid oxidation and oxidative phosphorylation reduce the M2 alternative marker arginase, in alternatively induced macrophages, concurrently with an abolishment of the anti-inflammatory effects of IL-4. PGC-1β interacts with STAT6 on the arginase I promoter to facilitate polarization towards an anti-inflammatory phenotype. Thus, the immune response is regulated by metabolic co-activators, which direct the macrophage towards use of fatty acid oxidation as an energy source, compared to the classically activated macrophages’ use of glucose [41]. Interestingly, oxygen consumption rates (OCR) were increased with lipids, but to a larger extent by PO than PA, indicating that PO-treated cells utilize fatty acid oxidation, with less reliance on glycolysis. This is in agreement with the fact that PO polarizes macrophages to the anti-inflammatory M2 phenotype [20].

The energy sensor, AMPK, is activated when the adenosine monophosphate (AMP) AMP: adenosine diphosphate (ADP) ratio increases in an attempt to restore energy. It activates fatty oxidation to generate ATP and inhibits unnecessary pathways, such as fatty acid synthesis [42]. When the tetrameric formation of AMPK is altered by deletion of the regulatory β1 subunit in macrophages, acetyl-CoA carboxylase (ACC) phosphorylation and mitochondrial content are affected, thereby inhibiting the action of AMPK and reducing fatty acid oxidation [43]. Nlrp3−/− mice on a HFD displayed increased fatty acid oxidation [28], as did ob/ob mice administered a caspase-1 inhibitor [5].

ROS can result from mitochondrial dysfunction and has already been previously discussed in relation to Nlrp3-inflammasome mediated IL-1β. Lipid peroxidation, a marker of oxidative stress, was positively correlated with BMI and waist circumference in obese humans. Multiple models of obesity increase oxidative stress, caused by increased ROS in the adipose tissue, regardless of their diabetic state. The mechanism at play was increased NADPH oxidase pathway and an impaired anti-oxidant response [44]. ROS can stabilize HIF-1α following LPS stimulation, another route by which it causes metabolic-inflammation [38]. ROS can also be generated two-fold in response to the SFA stearate stimulation in RAW 264.7 macrophage cells [30]. ROS production increases in adipocytes with increasing fat accumulation, with fatty acids (LA, arachidonic acid (AA), OA) causing the increase through generation of NADPH oxidase [44]. *In vitro* stimulation with ROS resulted in a dose-dependent decrease in adiponectin and increase in MCP-1 and IL-6. Inhibiting NADPH oxidase with Apocynin increases adiponectin, improves glucose and insulin sensitivity, reduces inflammation and decreases plasma triacylglycerol (TAG) levels, in obese, insulin resistant mice. Thus, anti-oxidants may have therapeutic potential in obesity-induced metabolic inflammation [44].

Metabolic switching is not unique to macrophages and it occurs during T cell differentiation and activation, as reviewed elsewhere [45,46]. AMPK is required for lymphocytes to adapt to mitochondrial stress. However, AMPK does not appear necessary for the metabolic switch which occurs in activated T cells whenmounting an immune response both *in vitro* and *in vivo* [47]. Interestingly, leptin [48] and fatty acid metabolism [49] are involved in T-cell responses, giving another example of how nutrition can influence the immune system [48].

### 2.5. Role of AMPK in Metabolic Inflammation

AMPK, a serine/threonine kinase, is an energy sensor which is implicated in inflammation [6], metabolism [42] and T2D [50]. It is responsible for adapting cellular metabolism in response to nutritional and environmental variations. Activated (phosphorylated) pAMPK is reduced in visceral, rather than subcutaneous, fat of obese humans, and is negatively correlated with inflammatory markers [51]. AMPK is also lower in insulin resistant, obese individuals (homeostatic model of insulin resistance (HOMA-IR) > 2.3), compared to BMI-matched counterparts [51]. In genetic mouse models of obesity, macrophage pAMPK expression was 33% lower than lean controls, with markedly increased TNF-α secretion [43]. In mice fed a MUFA-HFD, adipose pAMPK protein expression was not reduced despite obesity, compared to the SFA-HFD induced obesity.

Interestingly, our study demonstrated that AMPK activation was modulated by IL-1β activation in the adipose and bone marrow macrophage cells. Further *in vitro* investigation confirmed the involvement of this kinase in MUFA-mediated anti-inflammatory action [6]. PA and TNF-α incubation of endothelial cells lead to an increase in NF-κB, which is reduced with activation of AMPK through use of AICAR or overexpression of constitutively active AMPK [52]. LPS stimulation, fatty acids (palmitate/oleate/stearate) and acute lipid infusions significantly inhibited pAMPK and its downstream target ACC in macrophages [53]. AMPK induction with AICAR impeded LPS- and FFA-induced cytokine response, in part through NF-κB inhibition. AMPKβ1−/− BMDM display a predominant M1 profile, which was further exacerbated with PA and stearate treatment [43]. *In vivo*, AMPKβ1−/− ATM secrete more pro-inflammatory cytokines, which were further increased following a HFD. Increasing concentrations of PA up-regulate pAMPK, which was responsible for increased fatty acid oxidation as a method of buffering the PA-induced inflammation. Hematopoietic deletion of AMPKβ1−/− was sufficient to induce systemic inflammation in a HFD setting. AMPKβ1−/− contributes to insulin resistance with decreased pAkt, increased adipose non-esterified fatty acids (NEFA), hyperglycaemia and hyperinsulinemia [43]. Serum leptin levels increased and adiponectin reduced, indicating the negative effect of AMPK signaling disruption on adipose biology.

The concept of reversing HFD induced metabolic-inflammation is intriguing. BMDM derived from HFD mice retained a “dietary memory” with increased mRNA levels of Tnfα, Il6 and Nos2, compared to those of a LFD [20], which could be reversed with incubation of the *n*-6 MUFA, cis-palmitoleate (PO). This PO mediated anti-inflammatory effect was AMPK-dependent. To investigate the role of AMPK in the crosstalk between macrophages and adipocytes, a co-culture system between AMPKα1−/− macrophages with 3T3-L1 adipocytes was utilized. Insulin stimulated glucose uptake and insulin stimulated phosphorylation of insulin receptor were decreased when AMPK was deleted, confirming that the anti-inflammatory effects of macrophage AMPK can have positive effects on subsequent adipose biology [53].

In a similar manner to AMPK, inflammatory and lipid stimulation reduces another nutritional sensor, sirtuin 1 (SIRT1). SIRT-1 antagonized the LPS- and fatty acid-induced inflammation via impeded NF-κB signaling, with SIRT1 being a downstream signal from AMPK activation [42]. Metabolic inflammation can have paracrine effects on the neighboring organs which are instrumental in metabolic regulation.

## 3. Consequence of “Meta-Inflammation” in Metabolic Organs

### 3.1. Liver

The liver plays a central role in maintaining metabolic homeostasis, regulating the processes of lipogenesis, gluconeogenesis and cholesterol metabolism. However, obesity induced lipotoxicity and associated metabolic inflammation negatively impacts on hepatic lipid and glucose metabolism [54]. Lipotoxicity refers to the adverse effect of lipid accumulation on glucose and insulin metabolism [55]. Hepatic lipotoxicity develops when the liver exceeds its capacity to store and use fatty acids in the form of TAG. TAG stored in the liver are inert, however, when the liver’s storage capacity is exceed TAG are hydrolyzed back to fatty acyl CoA at a rate that exceeds the cells’ oxidative requirements [56]. This drives the formation of other potentially harmful lipid species such as ceramides, acyl CoAs, *etc.* [57]. Hepatic lipotoxicity may also reflect dysregulated fatty acid oxidation with the formation of ROS; disturbances in cellular membrane fatty acid and phospholipid composition; alterations of cholesterol content and through ceramide signaling [58]. Lipotoxicity drives the development of non-alcoholic fatty liver disease (NAFLD) by inducing a cascade of events including: hepatocellular death; activating Kupffer cells and an inflammatory response; and impaired insulin signaling; ultimately resulting in hepatic insulin resistance [58]. Hepatic insulin resistance is further driven by steatosis which impairs insulins ability to inhibit hepatic glucose production and stimulate glycogen synthesis [59]. Steatosis activates I kappa B kinase complex β (IKK-β) and NF-κB which upregulates IL-6 secretion [60]. IL-6 again in turn induces hepatocyte insulin resistance [61].

Excessive hepatic lipid accumulation is caused by an increase in endogenous fatty acid synthesis and FFA overflow from adipocytes into the liver [54]. The progression of NAFLD to non-alcoholic steatohepatitis (NASH) has been well reviewed elsewhere [62,63]. While there are numerous mechanisms implicated in the development of NASH, as in obesity, low adiponectin and increased TNF-α levels are hallmarks of the condition [63,64]. Hepatic lipotoxicity is an important pathophysiology associated with metabolic inflammation, which future therapeutics need to target. Therapies which improve insulin signaling and prevent the development of insulin resistance remain important strategies for reducing hepatic lipotoxicity and associated diseases such as NAFLD. Even moderate weight loss can result in improvements in hepatic insulin sensitivity [65].

### 3.2. Muscle

Skeletal muscle is responsible for approximately 80% of the glucose uptake in the body following insulin stimulation [66]. Therefore, conditions which impair insulin signaling, such as obesity induced metabolic inflammation, also impair skeletal muscle glucose metabolism. Lipotoxicity, with excessive intra-myocellular lipid accumulation, inhibits skeletal muscles ability to adequately respond to insulin signaling [67]. Again, lipid “overspill” from adipose tissue which has reached its storage capacity is stored in skeletal muscle in the form of TAG and other FFA. Excess FFA cause lipotoxicity and negatively impact on skeletal muscle insulin sensitivity [68]. The hypothesis that excess FFA induces insulin resistance is well established [69,70]. *In vitro*, treatment of myotubes with FFA decreases glycogen synthesis and glucose uptake and impedes insulin receptor signaling through Akt [71]. *In vivo*, lipid infusions drive skeletal muscle insulin resistance by decreasing insulin stimulated glucose metabolism and inhibiting IRS-1-associated phosphoinositide 3-kinase (PI3K) activity [69,72]. Itani and colleagues [73] show that acutely increasing plasma FFA levels during a hyperinsulinemic-euglycaemic clamp induces insulin resistance in human muscle. This induction of insulin resistance is associated with an increase in total membrane-associated PKC activity, translocation of the protein kinase C-β (PKC-β) and δ from the cytosol to the cell membrane, an increase in diacylglycerol (DAG) mass and a 70% decrease in the abundance of IκB-α, an inhibitor of NF-κB [73].

SFA derived from a HFD or LPS derived from the gut can impair insulin signaling through TLR4 to activate the NF-κB pathway [24,74] and prime the Nlrp3 inflammasome [16]. In this way, increased FFA plasma levels inhibit insulin signaling leading to reduced glucose transport activity which ultimately causes skeletal muscle insulin resistance [72]. Lipid induced insulin resistance in humans also results in alterations in DAG/PKC signaling [73]. Activation of PKC could induce insulin resistance through numerous mechanisms; one such mechanism is by increasing oxidative stress and activating the IKK-β/IκB-α/NF-κB pathway further driving inflammation and disrupting insulin signaling [73]. Other fatty acid derivatives, such as ceramides, also play a role in the development of insulin resistance. Obesity is associated with increased ceramide content in muscle which coincides with reduced insulin stimulated Akt phosphorylation [75]. Increased ceramide content can reduce insulin stimulated glucose uptake, the deleterious effects probably also reflect other FFA derivatives such as fatty acyl CoA or DAG [75].

Insulin signaling is not only important in terms of skeletal muscle glucose metabolism but also in terms of amino acid metabolism. Insulin also regulates muscle protein synthesis via activation of the mammalian target of rapamycin (mTOR) pathway [76]. Insulin resistance in skeletal muscle blunts its ability to adequately synthesize new protein in response to anabolic stimuli, such as amino acids [77]. Obesity impairs both skeletal muscle protein synthesis and whole body anabolic response to hyperinsulinemia and hyperaminoacidemia [78,79]. Stephens and colleagues [77] have shown that excess lipid availability can impede skeletal muscle glucose metabolism and amino acid metabolism, characteristic of insulin resistance and anabolic resistance, respectively. These findings were consistent irrespective of physical activity and diet-induced alterations in body composition. Overall DIO and the resulting state of chronic metabolic-inflammation negatively impact on skeletal muscle metabolism through a variety of mechanisms. Therefore, targeting the source of the problem—excess FFA and inflammation—seems an obvious approach, however, the best manner in which to target this approach is difficult to determine.

### 3.3. Pancreas

Similar to the liver and skeletal muscle, the pancreas is a metabolic organ negatively impacted by obesity induced lipotoxicity and glucotoxicity. Glucotoxicity in this case refers to the deleterious effect of chronic hyperglycemia on the pancreatic beta (β)-cells [80]. Obesity associated insulin resistance increases the metabolic demand on pancreatic β-cells. Excess FFA signal an increase in β-cell mass, insulin biosynthesis and insulin secretion in order to maintain normoglycemia and cellular homeostasis [81]. Eventually, the β-cells are unable to continue this compensatory mechanism and hyperglycemia ensues, driven by the elevated FFA levels [82]. The combined deleterious effects of glucotoxicity and lipotoxicity, referred to as glucolipotoxicity, eventually causes β-cell failure characteristic of T2D [82]. Chronic hyperglycemia as in obesity induced insulin resistance drives the development of glucotoxicity. Glucotoxicity results in a decrease in insulin gene expression in the pancreatic β-cells, characterized by a decrease in insulin synthesis and secretion [83]. ROS produced during glucose metabolism results in chronic oxidative stress which poses another potential mechanism for the development of glucotoxicity [84].

Interestingly, there seems to be a difference in the potential detrimental effects of different fatty acids on β-cell health. Elevated glucose and PA synergize to induce β-cell toxicity and caspase-3 mediated apoptosis [85] in a pancreatic cell line and isolated human islets [86]. Although linoleic acid (LA) induced cytotoxicity to some degree, this did not reach significance. Interestingly, OA was non-toxic, even at elevated glucose concentrations. In a similar study, but in human islets, PO or OA prevented PA/glucose induced β-cell death. OA and PO had other beneficial effects, increasing β-cell proliferation, reducing anti-apoptotic markers; increasing insulin content and secretory capacity of the β-cell [85]. OA and LA amplified glucose stimulated-insulin secretion through G-protein coupled receptor 40 (GPR40) [87]. *In vivo*, feeding a MUFA-HFD was capable of significantly increasing insulin secretion in response to a glucose challenge in a mouse model of obesity-induced insulin resistance compared to SFA-HFD [6]. Glucose was shown to inhibit fatty acid oxidation in islets, indicating that partitioning toward oxidation and away from esterification may be a protective mechanism [86]. Activation of AMPK with either metformin or AICAR inhibited the PA-induced cell death, demonstrating another of the pleiotropic roles that AMPK has within fatty acid induced metabolic dysfunction [86].

Islet amyloid polypeptide (IAPP) is a peptide secreted from the pancreas with insulin, which forms aggregates in T2D and can have inflammatory consequences. Human amyloidogenic IAPP activates the Nlrp3 inflammasome to release pro-inflammatory IL-1β from immune cells present in the pancreatic islets [88]. Sufficient glucose metabolism is required for the priming step in this process, as 2-deoxy-d-gluocse (2-DG) treatment inhibited the IAPP-induction of IL-1β. HFD feeding in a mouse model with a transgenic form of IAPP confirmed that it could activate IL-1β in an *in vivo* setting [88]. Nlrp3 was further implicated in causing obesity-induced pancreatic damage in a mouse model of obesity [89]. Interestingly, Nlrp3−/− and ASC−/− mice on a HFD for one year had increased insulin levels but were still protected against insulin resistance, displaying β-cell compensatory protective mechanisms at play. Nlrp3 also had roles in causing islet fibrosis and β-cell death, common occurrences in obesity-induced pancreatic damage. Given the important role that obesity and different dietary constituents play in causing obesity associated metabolic-inflammation, we next describe the various therapeutic and dietary changes that can be used to improve the phenotype in humans.

## 4. Therapies to Improve Inflammation and Metabolic Health

### 4.1. Pharmaceuticals That Target Metabolic Inflammation

According to the World Health Organization (WHO) 2014 report [90], 600 million people worldwide are obese. This translates to enormous healthcare costs worldwide for obesity and its related diseases. Therefore, potential therapeutics are at the forefront of obesity research. Metformin is widely used in the prevention and treatment of T2D, following a 31% reduction in the incidence of T2D in a large cohort of 3234 individuals in a clinical trial study compared to the placebo group [50]. Metformin reduces fasting glucose levels via suppression of endogenous glucose production. Metformin is also an activator of AMPK, which also explains its efficacy. However, a lifestyle intervention lowered the incidence of T2D by 58% compared to placebo, making it potentially more effective than Metformin treatment [50]. Another target of AMPK signaling is berberine (BBR), a major form of isoquinoline alkaloid derived from medicinal herbs. BBR treatment reduced the expression of PPARγ, TNF-α and IL-1β in the adipose and SVF of WAT from db/db mice [30]. BBR-stimulated macrophage conditioned media, despite LPS stimulation, improved insulin stimulated glucose uptake in adipocytes. Inhibition of the MAPK pathway, ROS and NO generation and AMPK activation are the methods used by BBR to mediate its anti-inflammatory effects in metabolic disorders [30]. In a meta-analysis of human interventions using BBR, it was found to be more effective at reducing T2D and hyperlipidemia in conjunction with a lifestyle intervention, compared to lifestyle interventions alone [91]. The authors advised caution in their conclusions as not all studies had optimal study design or adequate numbers in the cohort.

Thiazolidinediones (TZD) are a class of insulin sensitizing drugs which are selective ligands of PPARγ and target insulin resistance [92]. TZDs exerted their beneficial effects on glucose metabolism by increasing peripheral glucose disposal and adiponectin secretion, while ameliorating inflammation by decreasing FFA and pro-inflammatory cytokine levels [93]. However, the negative side effects associated with TZDs have limited their use; highlighting the need for alternative PPAR ligands to be identified. Currently, a range of PPARγ activating natural products, described by Wang and colleagues [94], are being investigated for their potential therapeutic efficacy. While the effects of the natural PPARγ ligands investigated to date are not as potent as the TZDs [94], this is an area which warrants further research.

Despite its original use in the treatment of inflammatory conditions such as rheumatoid arthritis [95], the observation that salicylates have hypoglycemic effects caused it to be reinvestigated as a potential therapy for insulin resistance and T2D [96]. Salicylate inhibits IKKβ, to prevent the deleterious effects of excess FFA on insulin signaling and action [95,97]. Therefore, while the anti-inflammatory potential of nutrients such as polyphenols may be difficult to translate in the clinical trial setting, the success of salicylate highlights the potential of therapies which target IKKβ/NF-κB inhibition in terms of attenuating metabolic-inflammation and insulin resistance [60,98].

Anakinra is an IL-1R antagonist used in the treatment of T2D, due to its ability to lower glucose levels. Despite increased β-cell function and anti-inflammatory effects, no improvement in insulin sensitivity was observed and, thus, it is not a complete therapy for obesity induced insulin resistance [99]. Glyburide is a sulfonylurea which lowers blood glucose by increasing insulin release from the pancreas and is therefore used in the treatment of T2D [100]. Glyburide is also an inhibitor of the Nlrp3 inflammasome [101], an attractive target for metabolic-inflammation, given the detrimental effect it has in both inflammation and insulin resistance. Other anti-inflammatory therapies, such as anti-TNF, are commonly used for chronic inflammatory diseases, such as rheumatoid arthritis [102], but without an insulin sensitive effect. Due to the necessary role that TNF-α has in our defense system, infections and adverse effects are commonplace with anti-TNF therapies [103]. Pharmaceutical therapies provide a proof of concept as to the treatment of T2D and identify mechanisms by which they act. Nutrients are capable of modulating similar pathways and are under investigation as potential treatments for obesity-associated complications.

### 4.2. Weight Loss and Improved Insulin Sensitivity

Weight loss is the most effective lifestyle intervention to improve insulin sensitivity and preserve β-cell function. It can also inhibit progression of insulin resistance to overt T2D. Significant energy restriction diets (600 kcal/day diet) improve β-cell function and insulin sensitivity, associated with reduced hepatic and pancreatic TAG stores, in T2Ds [104]. Weight loss of greater than 5%, reduces sub-acute inflammatory markers [105]. The Finnish Diabetes Prevention Programme showed that lifestyle interventions which target individuals with impaired glucose tolerance and increased risk of T2D can prevent or delay the development of T2D [106]. While lifestyle interventions such as this are of benefit; this approach has limited efficacy in terms of long-term compliance. It is estimated that 22 individuals would need to be treated with this approach for one year to prevent the development of one case of diabetes [106]. However, weight loss is difficult to achieve and maintain; therefore, pharmacotherapies or nutritional strategies need to be explored in combination with lifestyle interventions [107]. The glucoregulatory incretin glucagon like peptide-1 (GLP-1) increases satiety. The use of GLP-1 analogues to prolong GLP-1 receptor activation is associated with weight loss [108]. A recent large scale study showed that a GLP-1 analogue liraglutide (3.0 mg/day) in combination with lifestyle modification reduces body weight and improves metabolic health [109]. Surgical interventions, such as Roux-en-Y gastric bypass or biliopancreatic diversion, which results in weight loss, also improve insulin sensitivity in obese individuals [110]. However, these invasive therapies are generally restricted for use in individuals with serious co-morbidities or in whom lifestyle and medical weight loss interventions have failed.

### 4.3. Manipulation of Dietary Fat

As we have established throughout this review, SFA is detrimental to health, while MUFA and PUFA may have less adverse and/or protective effects. This potential paradigm is relatively consistent *in vitro* and in animal DIO models. The Mediterranean diet has a characteristically high MUFA content, derived from olive oil and nut consumption, with high fiber and low red meat intake. Several southern European studies suggest beneficial effects of this dietary pattern. The ATTICA study took place in Greece and participants with a diet score in the highest tertile based on adherence to the Mediterranean diet had reduced inflammation and coagulation markers [111]. The CORonary Diet Intervention with Olive Oil and Cardiovascular PREVention (CORDIOPREV) is a dietary intervention whereby individuals with T2D are given either a standard healthy diet or a Mediterranean style diet. Whilst the adverse effects of dietary SFA remain consistent, the beneficial effects of MUFA are not as uniform in northern European cohorts. Cross sectional studies show that habitual SFA intake is associated with HOMA-IR and inversely associated with the insulin sensitivity index (ISI). The KANWU study investigated the effect of SFA *versus* MUFA in obese men and showed that SFA impaired insulin sensitivity and MUFA did not improve insulin sensitivity [112]. Similarly, replacement of SFA with MUFA or a low fat high complex carbohydrate (LFHCC) diets did not improve insulin sensitivity in individuals with the Metabolic Syndrome (MetS) [113], although the incidence of the MetS was reduced [114]. Whilst high SFA consumers had higher adipose caspase-1 mRNA levels, reducing dietary SFA had no effect on inflammation [113].

Nevertheless, there are interesting insights from *post-hoc* analysis of these studies. The 16-week MUFA intervention improved HOMA-IR, but only in those with a low habitual dietary fat intake. Also, the pre-intervention insulin resistance state determines whether an individual responds to an intervention or not [114]. From a mechanistic perspective, there are a few possibilities. Feeding a SFA HFD is associated with a hypertrophic adipose morphology, whereas feeding a MUFA HFD is associated with an equal adipose weight but a hyperplastic morphology, coincident with insulin sensitivity [6]. However, if a SFA-HFD is fed before the MUFA-HFD, it is not possible to revert the hypertrophic to hyperplastic adipose phenotype. This suggests that SFA and MUFA may differentially affect adipogenesis which is irreversible in later life; therefore, early diet or habitual dietary priming may be an important determinant of dietary efficacy. Long chain *n*-3 PUFA (LC *n*-3 PUFA) may have benefit over SFA. Cellular, animal and cross sectional human data is promising. Oliver and colleagues [115] extensively reviewed the potential use of LC *n*-3 long PUFA in reducing inflammation and macrophage accumulation in T2D. Despite the promising anti-inflammatory effects of PUFA *in vitro* [31] and that cross-sectional studies show potential, the human intervention results to date are not positive. The Professionals Follow-up Study investigated the associations between *n*-3 PUFA and health, and demonstrated that high intakes of both EPA and DHA were inversely correlated with levels of soluble TNF-1 and TNF-2 receptors [116]. However, dietary supplementation of low doses of LC *n*-3 PUFA, in conjunction with a low-fat, high complex carbohydrate, does not improve IS in MetS subjects [113]. Overall, manipulation of dietary fat quality, in terms of reducing SFA, is a possible avenue for positive manipulation of metabolic inflammation.

### 4.4. Supplements/Functional Foods

Given the pivotal role of NF-κB in driving inflammation in response to the cellular stressors associated with obesity, therapies which target this transcription factor are of clinical importance. Supplements and functional foods may offer nutritional approaches to deliver the therapeutic benefit of inhibiting NF-κB with minimal adverse outcomes. Polyphenols such as epigallocatechin gallate (EGCG) impede NF-κB activation by blocking IKK activity [117] and have been show to attenuate the development of obesity and its associated co-morbidities such as insulin resistance in a DIO mouse model [118]. While EGCG has shown positive results in terms of obesity markers *in vitro* and *in vivo*, the human studies carried out to date show inconsistencies in its effectiveness [119]. Other polyphenols such as resveratrol [120] and curcumin [119] also inhibit NF-κB and have potential anti-inflammatory and anti-oxidant effects which may be used to attenuate obesity associated chronic inflammation. However, similarly to EGCG, the promising *in vitro* and *in vivo* animal data have been difficult to translate to improved patient outcomes in clinical trials for both resveratrol [121] and curcumin [119]. While the anti-inflammatory effects of these polyphenols may be difficult to translate to human studies, the strong anti-inflammatory effects observed *in vitro* and in animal studies highlight their potential as therapeutics in chronic inflammation, as in obesity. Combinations of anti-inflammatory dietary products have been shown to modulate inflammation and metabolic stress in overweight individuals [122]. This may offer a way in which the anti-inflammatory potential of polyphenols may be enhanced in order to improve their clinical efficacy.

## 5. Future Perspectives and Conclusions

As discussed throughout this review, metabolic-inflammation in the metabolic and immune cells of adipose, liver, pancreas and skeletal muscle contributes to the development of obesity induced insulin resistance and T2D. Hypertrophic adipose tissue occurs in obesity with reduced adipogenesis and the inability to maintain insulin sensitivity. Initial immune cell infiltration is a protective mechanism, but with increasing adiposity, immune cell number and chemokine secretion proportionally increase. The immune cells undergo a phenotypic switch from M2 anti-inflammatory to M1 pro-inflammatory, with the Mme in between the two. SFA, namely PA, induce the former, with MUFA and PUFA influencing the latter phenotypes. Inflammatory mediators can inhibit insulin signaling and glucose transport and worsen the already established inflammation within the metabolic tissues. TNF-α and IL-1β are two of the major players in this event. Inflammation and diet combined can determine the metabolic pathway utilized by the cell. Inflammation undergoes metabolic reprogramming, with a switch from energy efficient oxidative phosphorylation to the less efficient glycolysis. Downstream metabolites from this can then feedback and cause further inflammation and oxidative stress. The energy sensor, AMPK, has been shown to be involved in metabolic fatty acid oxidation, while having an anti-inflammatory effect induced by OA and PO. Conversely, pAMPK is decreased in obesity and with SFA. Lipid “spillover” from the expanding adipose tissue ends up causing lipotoxicity and hepatic steatosis within the liver, which is ill equipped to deal with excess FFA. The skeletal muscles’ inability to respond effectively to insulin is a direct result of lipid accumulation within this tissue. Furthermore, PA can activate the NF-κB pathway exacerbating the situation. The pancreas is sensitive to hyperglycemia and develops glucotoxicity as a result. This can lead to β-cell dysfunction and eventual failure, with the pancreas being the instrumental organ in insulin secretion. Obesity *per se* is a nutritional stressor at the heart of the metabolic-inflammatory environment. Lifestyle interventions and weight loss are effective but difficult to maintain. There are many therapeutics available, however, their incomplete effects or side effects mean there is no one cure for all the symptoms of metabolic-inflammation and insulin resistance. Dietary manipulation of fat quality is an attractive option but mixed results make it hard to enforce.

Discrepancies between *in vitro*, animal and human studies make it difficult to ascertain the exact mechanisms at play and, more importantly, how best to treat them. Although *in vitro* and animal studies provide an opportunity for mechanistic examination of the pathways involved using genetic deletion, inhibition with drugs, treatments with individual dietary components and use of elaborate techniques; these are not all possible in human studies. The lack of translation between these models and humans is understandable; however, the lack of consistency and findings among human studies is harder to accept. Various factors including differences in study design, doses of nutrients and drugs utilized, combination of nutrients within a human diet, lifestyle, and many other aspects could be confounding the results from human intervention studies. The interplay between different fatty acids, inflammatory cytokines, metabolic pathways and nutrient and pathogen sensing pathways further complicate the field. Further investigation is required to decipher if dietary fatty acids affect the metabolic switch in immune cells and how metabolites can affect the immune and metabolic tissues. Metabolism and inflammation have not yet been effectively demonstrated in human studies and warrant further research. More combination studies are required as different inflammatory mediators interact and synergize with one other, the same of which is true for dietary constituents. Regression of the insulin resistant phenotype needs more attention as, realistically, this is the scenario we are attempting to address in the human setting. The complexity of all these pathways in obesity leading to adipose tissue expansion, lipotoxicity, glucotoxicity, inhibition of insulin signaling, and low-grade chronic inflammation means there are multiple sites that require targeting. Given the specificity, high cost and adverse effects of pharmaceuticals, perhaps, nutrient therapies are the better option. Nutritional interventions allow for easier combination therapies, with fewer side effects, and also allow for longer term treatment. A whole body approach is required which involves weight loss, and use of anti-inflammatories, insulin sensitizers and anti-oxidants in order to fully combat obesity-induced metabolic-inflammation and its subsequent diseases.
